# Posterior transforaminal debridement and interbody fusion with instrumentation for multi-segment thoracic spinal tuberculosis: a midterm follow-up study

**DOI:** 10.1038/s41598-022-23169-x

**Published:** 2022-10-29

**Authors:** Zhenchao Xu, Zhen Zhang, Yunqi Wu, Xiyang Wang

**Affiliations:** 1 Department of Spine Surgery and Orthopaedics, The Xiangya Hospital of Central South University, 87# Xiangya Road, Changsha, 410008 Hunan China; 2 Hunan Engineering Laboratory of Advanced Artificial Osteo-Materials, 87# Xiangya Road, Changsha, 410008 Hunan China

**Keywords:** Neuroscience, Diseases, Medical research

## Abstract

This retrospective study aimed to evaluate midterm outcomes of surgical management of multi-segment thoracic spinal tuberculosis by single-stage posterior transforaminal debridement and interbody fusion with instrumentation. From January 2007 to October 2015, 42 adult patients with thoracic spinal tuberculosis involving three or more levels underwent single-stage posterior transforaminal debridement, interbody fusion and instrumentation At a mean follow-up of 73.5 ± 9.6 months, all patients were eligible for final evaluation. All displayed improved biochemical markers and pain scores at 3 months and improved physiologic levels at the end of treatment. Visual analogue and 36-Item Short-Form Health Survey scores were significantly improved compared with preoperative values. All 30 patients with preoperative neurological deficits experienced neurologic improvement. Thoracic kyphosis angle decreased significantly from 34.4° ± 4.5° to 22.0° ± 2.6°. A mean kyphotic angle loss of 1.7° ± 1.1° was recorded at the final follow-up, and bone fusion was observed at a mean of 10.6 ± 2.1 months, with no instrumentation failures. One patient experienced delayed incisional healing and five patients suffered postoperative intercostal neuralgia that were cured by conservative treatment. There were no graft failures or implant breakages. This study showed the utility of a single-staged procedure combining posterior transforaminal debridement and interbody fusion with instrumentation, and demonstrated promising results.

## Introduction

Skeletal tuberculosis (TB) imposes a formidable disease burden, especially in the developing world^1^. Significant challenges in spinal TB arise from osteolysis that may cause deformity, neurological deficit, and even paraplegia^2^. Anti-TB chemotherapy remains the mainstay of treatment for spinal TB^3^. Options include drug therapy alone, surgery followed by medical therapy, or concomitant use of both modalities^4,5^. Conservative medical management cannot fully prevent the potential progression of kyphosis, which often results in chronic back pain, residual deformity, or both^6^. Although its incidence is relatively low, multi-segment thoracic spinal TB can cause acute symptoms and disability. Consequently, treatment is difficult, and surgical procedures are indicated more often than for single-segment thoracic spinal TB^7,8^. The lack of a consensus-based management protocol for these infections has been noted in the literature. The central segment of the vertebral body often develops extensive osteolysis with sequestrum fragments and massive cold abscesses, which can easily invade the spinal canal^9^. The location of vital organs near the thoracic spine, confounds surgery for multi-segment thoracic spinal TB. Adjacent organs should be protected from injury, and attention should be paid to the restoration of spinal stability after the lesion is cleared.

With the development of advanced treatments and surgical options, stabilization through posterior instrumentation remains the mainstay for the correction of angular deformities and provides stabilization; however, fusion across spinal segments has been reported when the anterior approach is indicated for easier access to diseased segments of vertebral bodies^10,11^. Transforaminal thoracic interbody fusion aims to avoid retraction of neural elements that is inevitable during the anterior approach^12^. Due to a lack of evidence-based guidelines for optimal treatment and management strategies, the therapy of multi-segment thoracic spinal TB remains controversial. We present our experience with a midterm follow-up of management that uses a single-stage debridement through the posterior transforaminal approach coupled with stabilization using fusion and instrumentation.

## Methods

### Patient diagnosis, inclusion, and exclusion criteria

**Figure 1:**
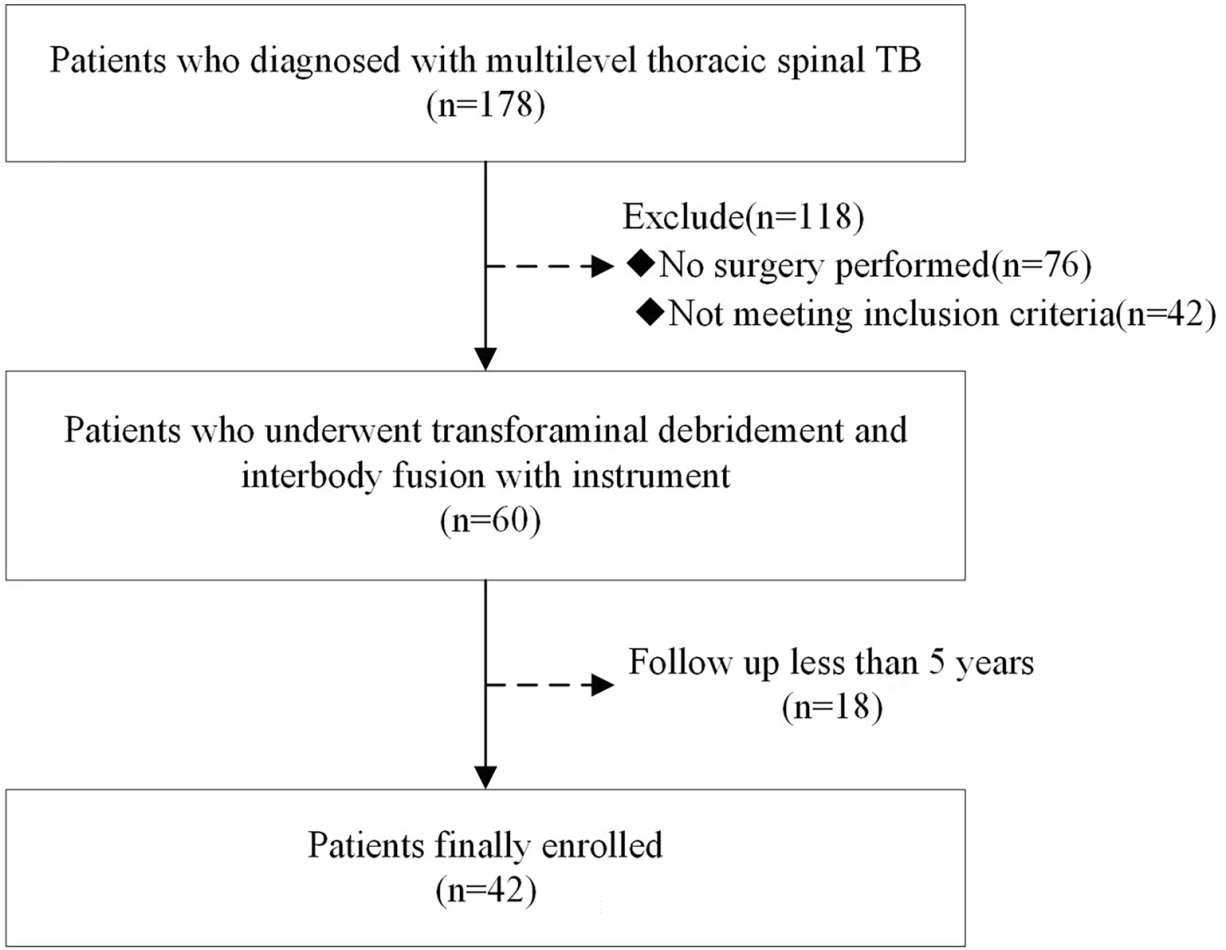
Flow chart of the patients meeting inclusion and exclusion criteria.

From January 2007 to October 2015, 178 patients with multi-segment (three or more level involvement) thoracic spinal TB were evaluated at our center. In total, 76 patients did not undergo surgical management, and 42 patients were not candidates for a single-stage posterior approach procedure due to clinical characteristics (exclusion criteria are listed below). In total, 60 patients underwent a single-stage posterior approach debridement through the transforaminal corridor and stabilization using interbody fusion as well as instrumentation. A total of 42 patients were eligible for final evaluation at a minimum 5-year follow-up and were included in this study (Table 1) and 18 patients were lost to follow-up before completion of treatment or before the study endpoints were achieved (Fig. 1). Patients with the following surgical indications were included: (i) vertebral lesions that caused spinal instability and chronic back pain; (ii) progressive neurologic dysfunction caused by spinal cord compression; (iii) unpreventable progressive kyphosis, evidenced by an increasing thoracic kyphosis angle or Tuberculosis Spine Instability Score^13^; and (iv) no pain relief or progressive vertebral destruction after anti-TB therapy, indicating poor response to conservative treatment. Exclusion criteria were (i) large paravertebral abscess or gravity abscess formation; (ii) previous thoracic spinal surgery (thus at risk for abnormal regional anatomy); and (iii) limited osteolysis and abscess, with neither spinal cord compression nor spinal instability. Twenty-five patients were male and 17 were female. The average age at the time of surgery was 45.8 ± 13.8 years. Lesions involved three levels in 19 patients, four levels in 14 patients, five levels in eight patients, and six levels in one patient. Of these, 11 patients exhibited skip lesions. All patients presented with at least one constitutional symptom, such as back pain, weakness, malaise, night sweats, low-grade fever, or weight loss. Disease-specific presentations were noted in 22 patients (52.4%) with intercostal neuralgia, 30 patients (71.4%) with paraparesis, and one patient (2.4%) with paraplegia. Initial working diagnoses of TB were based on biochemical and radiological parameters. Diagnoses were confirmed by histopathologic findings or a culture of tissue samples that yielded *Mycobacterium tuberculosis*.

**Table 1:** Demographic, disease characteristic and operative information of patients (average ± standard deviation).

Number of patients	Gender		Age (years)	Duration of symptoms (months)	Intraoperative blood loss (mL)	Operation time (min)	Follow-up period (months)
	Male	Female					
42	25	17	45.8 ± 13.8	5.3 ± 2.0	842.9 ± 182.7	182.2 ± 26.0	73.5 ± 9.6

### Preoperative procedures

All patients received standard anti-TB chemotherapy as advised by the institutional protocol comprised of isoniazid, rifampicin, ethambutol, and pyrazinamide. Doses were adjusted to patient weight with considerations for maximum daily doses before the initiation of treatment. Surgery was scheduled after recovery from anemia and hypoproteinemia as well as a reduction of inflammatory marker levels following medical therapy. Immediate surgery was indicated only if a patient experienced acute paralysis or progressive neurological impairment during medical therapy.

Patients underwent preoperative assessments of biochemical markers, erythrocyte sediment rate (ESR) and c-reactive protein (CRP) levels, neurological function (American Spinal Injury Association [ASIA] impairment scale), Visual Analog Scale (VAS) pain scores, quality of life (36-Item Short-Form Health Survey questionnaire [SF-36 scale] scores), and measurements of thoracic kyphotic angles.

### Operative procedures

**Figure 2:**
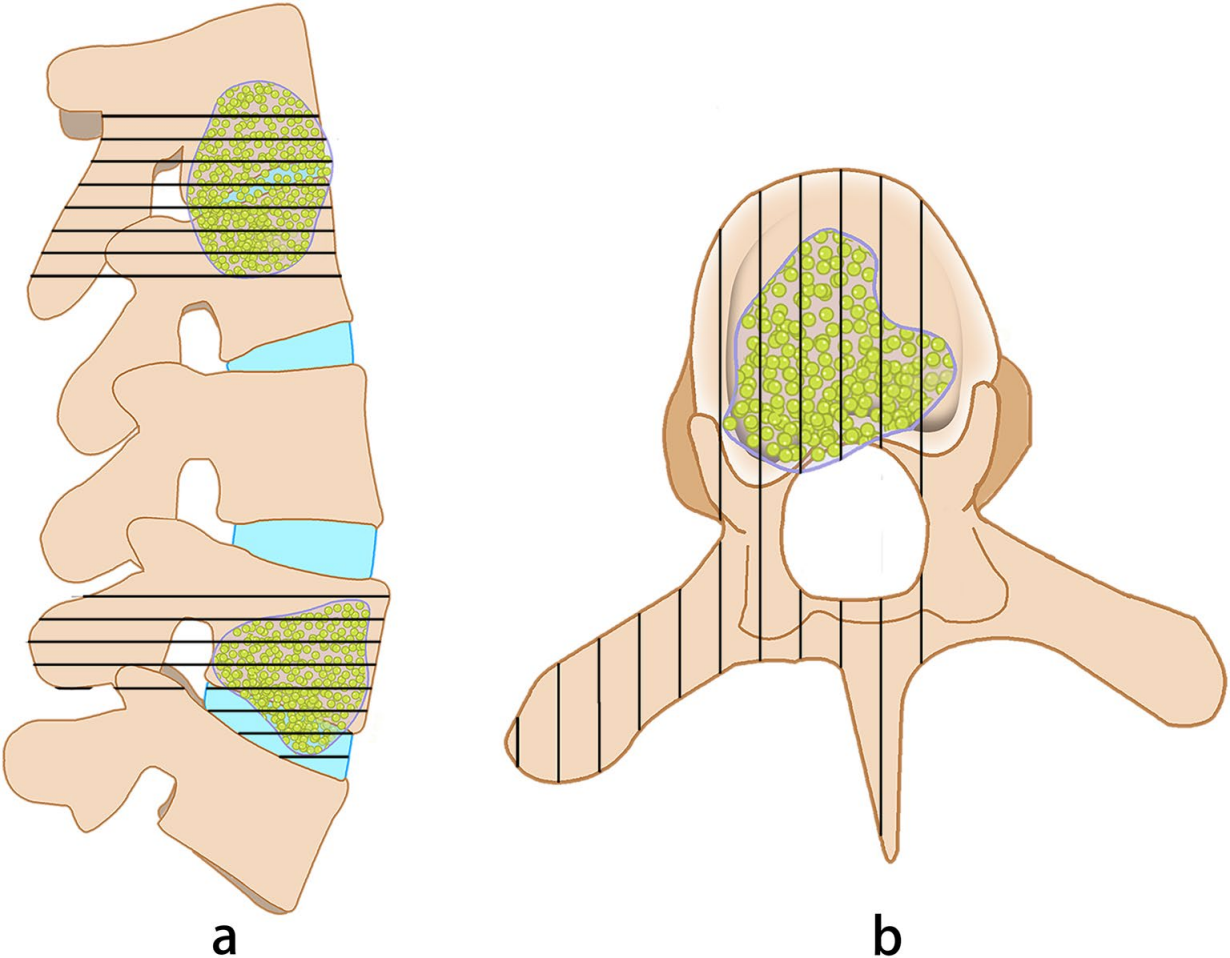
A posterior approach for multi-segment thoracic spinal TB. Black striped areas show the range of excision including spinous processes, unilateral (or bilateral) facet joints, pedicle of vertebral arch and transverse process in both sagittal (**a**) and transverse (**b**) planes.

Each patient was placed in a prone position after the administration of general anesthesia. A soft cushion was placed under the chest and pelvis so that the abdomen could hang freely, which reduced kyphosis. For patients undergoing an upper thoracic spine procedure, intraoperative halo traction was performed to reduce kyphosis. The diseased segment was localized under an image intensifier and a posterior midline approach was centered over the affected levels. Posterior elements including lamina, facet joints, and transverse processes were exposed (subperiosteal dissection at the fusion levels, extraperiosteal dissection at non-fusion levels), followed by dissection at the level of decompression and extended bilaterally to expose costotransverse articulations. Subsequently, the placement of screws was performed. Generally, longer segmental fixation was preferred, between at least two levels superior and inferior to the level of decompression. Affected vertebral bodies with limited destruction of the disc and adjacent vertebral bodies (less than half of the vertebral body destroyed as adjudged on plain lateral radiograph) were often incorporated into the instrumentation system. A hemi-laminectomy or laminectomy was conducted on the most severely involved aspect of the diseased segment. Exposure was increased as appropriate by sacrificing the thoracic nerve roots on the most affected side to allow a more thorough debridement when offering a > 270° decompression (Fig. 2a,b). Before destabilizing the vertebral column, temporary rod stabilization was utilized on the aspect with milder osteolysis. This allowed disease clearance and avoided spinal cord injury from instability. We drained prevertebral abscesses and exposed diseased vertebral bodies. Lesions including vertebral sequestra, diseased intervertebral discs, cold abscesses, and granulation tissue were removed completely by curettage through to healthy bleeding bone for spinal cord decompression, preferably with angled curettes and disk forceps protecting the spinal cord. It should be noted that the spinal cord was not stretched or distracted. The extent of the interbody fusion was determined and the endplate was debrided to achieve bone surfaces to facilitate fusion. A suitable autograft block from ilium or allogeneic bone block was used for fusion. Local installation of streptomycin and isoniazid (1.0 g and 0.2 g, respectively) was utilized before closure over a suction drain. All diseased tissue was collected for histopathologic examination and microbial culture assessment.

### Postoperative procedures

Postoperative orthogonal radiographs were taken to document decompression and instrumentation. Patients underwent postoperative in-bed physical therapy while maintaining a supine position for an average of 4 weeks. Out-of-bed mobilization was initiated gradually, and orthosis was used as required. Anti-TB therapy was continued postoperatively for 12–18 months as recommended by the institutional protocol.

### Follow-up

Regular outpatient reexaminations and assessments of spinal parameters were conducted every 3 months during the first postoperative year and every 6 months thereafter. A follow-up duration of at least 5 years was carried out for each patient. At each follow-up, ESR, CRP, ASIA impairment scale, VAS pain scores, SF-36 scale scores, and thoracic kyphotic angles were recorded. Radiographs were advised at routine follow-up for assessment of implant status. Bone fusion status was assessed according to the modified radiologic criteria by Lee et al.^14^.

### Statistical analyses

Statistical analysis was performed using SPSS version 18.0 (IBM Corp, Armonk, NY, USA) for descriptive data as well as comparative analysis using a paired *t* test, which was utilized to assess the difference in study parameters (preoperative versus postoperative) with a two-tailed p-value significant at *p* < 0.05.

### Ethics approval and consent to participate

This study protocol was approved by the Ethics Committee of Xiangya Hospital Central South University, We also followed the Declaration of Helsinki and its later amendments. Written informed consent was obtained from all patients.

## Results

In total, 42 patients with a mean follow-up of 73.5 ± 9.6 months were analyzed at the final follow-up. All patients had pathologic diagnoses of TB demonstrated by either tuberculous granulomas or caseous necrosis. Cultures from 11 patients yielded *M. tuberculosis*. None of the isolates were drug-resistant.

The average intraoperative bleeding volume and surgical case duration were 842.9 ± 182.7 mL and 182.2 ± 26.0 min, respectively. The average pretreatment ESR and CRP were 60.6 ± 18.6 mm/h and 43.9 ± 11.2 mg/L, which significantly decreased to 11.4 ± 2.7 mm/h and 4.4 ± 1.2 mg/L 3 months after surgery, respectively. Furthermore, at the final follow-up, ESR and CRP values returned to 4.2 ± 1.5 mm/h and 1.8 ± 0.7 mg/L respectively. Preoperative ESR and CRP values were significantly higher (*p* < 0.05) than those obtained at the 3-month postoperative and final assessments.

The mean VAS score was 7.0 ± 1.2 preoperatively and decreased to 0.8 ± 0.7 at the final follow-up (Table 2). Postoperative quality of life was significantly improved compared with pre-operative values according to SF-36 scale scores (Table 3).

**Table 2:** Clinical evaluation indices of the preoperative and postoperative follow-up (average ± standard deviation).

	Pre	TMP	FFU
ESR (mm/h)	60.6 ± 18.6	11.4 ± 2.7*	4.2 ± 1.5*
CRP (mg/L)	43.9 ± 11.2	4.4 ± 1.2*	1.8 ± 0.7*
VAS	7.0 ± 1.2		0.8 ± 0.7*
Thoracic kyphosis angle (°)	34.4 ± 4.5	22.0 ± 2.6*	23.7 ± 2.1*

*Analyzed by paired *t* test, compared with preoperatively, *p* < 0.05.

*Pre* preoperative, *Post* postoperative, *TMP* 3 months postoperative, *FFU* final follow-up.

**Table 3:** Quality of life according to SF-36 scale (average ± standard deviation).

	Pre	FFU
Physical functioning	64.5 ± 5.3	80.2 ± 2.9*
Role (physical)	15.9 ± 8.1	79.9 ± 10.9*
Bodily pain	42.2 ± 7.7	78.8 ± 10.3*
General health	24.0 ± 7.9	76.1 ± 8.1*
Vitality	43.1 ± 9.3	77.1 ± 8.8*
Social functioning	23.9 ± 9.4	75.2 ± 8.2*
Role (emotional)	28.4 ± 9.8	78.3 ± 8.6*
Mental health	23.7 ± 9.5	77.8 ± 8.6*

*Analyzed by paired *t* test, compared with preoperatively, *p* < 0.05.

*Pre* preoperative, *FFU* final follow-up.

The ASIA impairment scale recorded preoperative neurological deficits (Table 4) in 30 patients, of whom 25 regained normal neurologic function at the final assessment. The remaining five patients had partial improvement in neurological grade. No post-operative exacerbations of neurological deficits occurred.

**Table 4:** Neurological functions according to the ASIA impairment scale for preoperative and postoperative follow-up.

Pre		FFU				
		A	B	C	D	E
A	1			1		
B	1				1	
C	3				1	2
D	25					25
E	12					12

*Pre* preoperative, *FFU* final follow-up.

**Figure 3:**
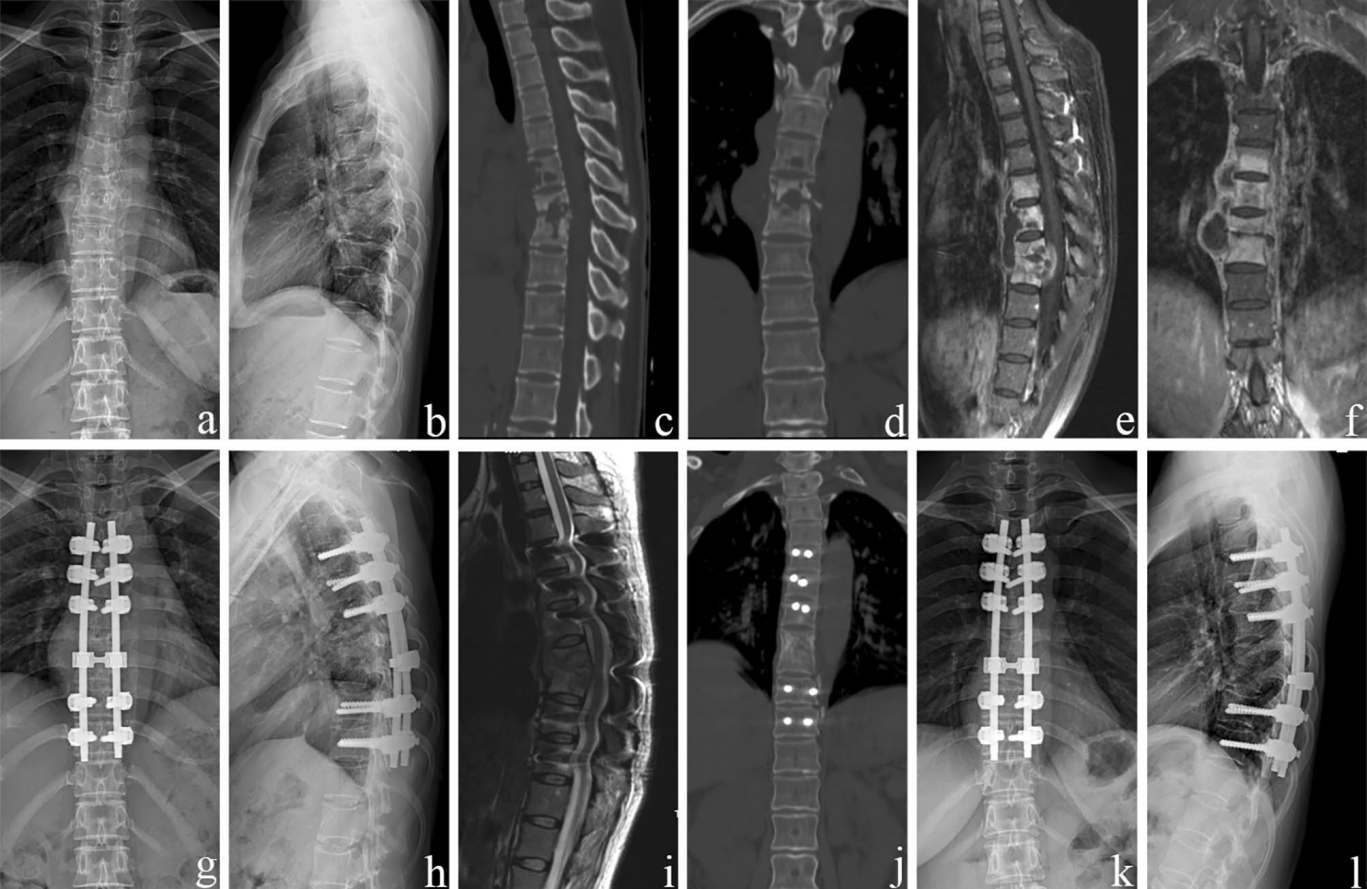
A 49-year-old female demonstrating lesions received a one-stage posterior transforaminal thoracic debridement, interbody fusion, and instrumentation. (**a**–**f**) Preoperative radiographs, CT, and MRI scans show TB of T5–T8 with vertebral bone destruction and deformity (kyphosis angle 36°) and paravertebral abscess formation. (**g**,**h**) Postoperative radiographs demonstrate the correction of the deformity (kyphosis angle 23°). (**i**) Postoperative MRI that shows clearance of TB lesions and spinal cord decompression. (**j**) CT shows satisfactory bone fusion at 12 months. (**k**,**l**) Radiographs display good internal fixation position and solid bone fusion, with a correction loss of 1° after 81 months of follow-up.

**Figure 4:**
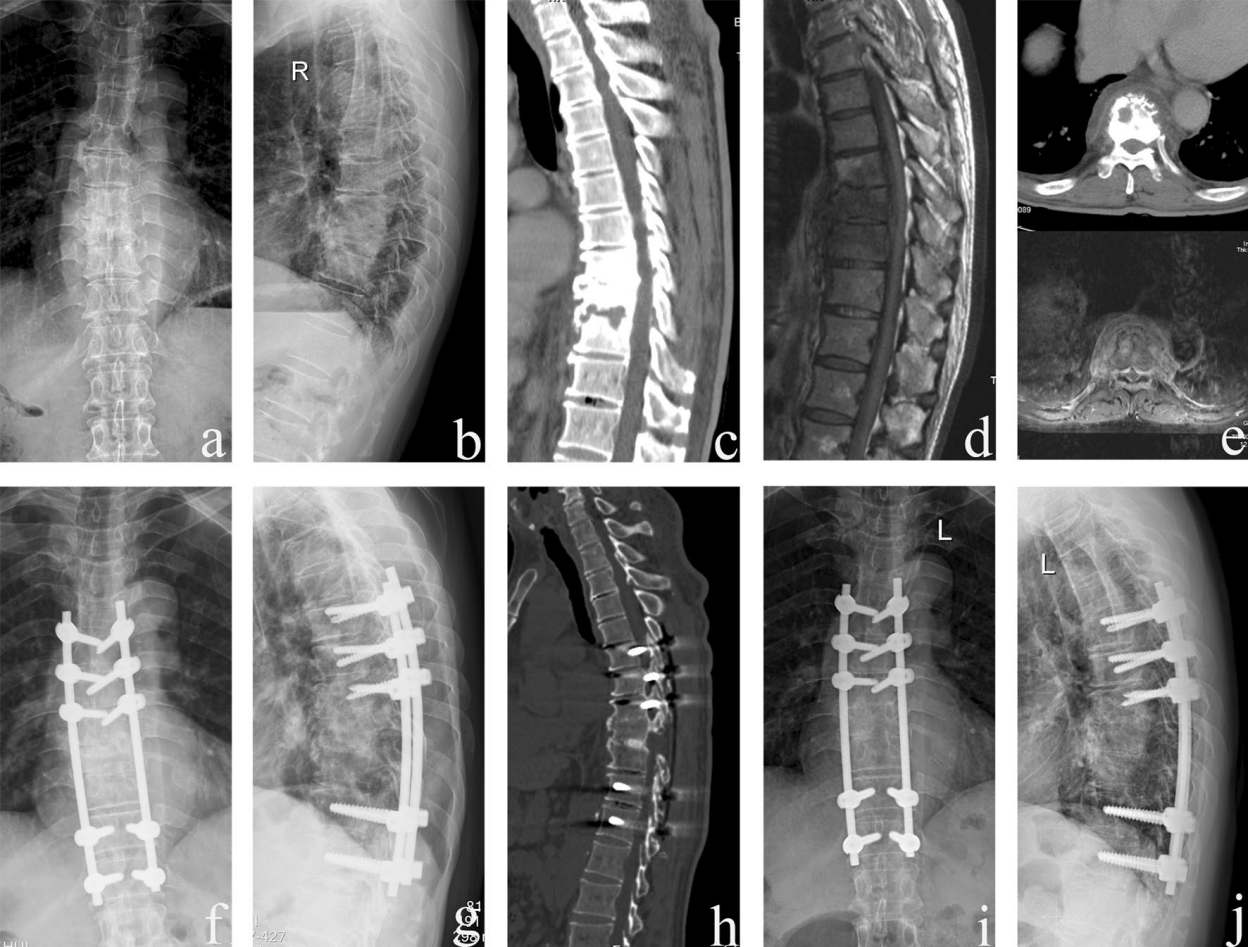
A 57-year-old male received a one-stage posterior transforaminal thoracic debridement, interbody fusion, and fixation. (**a**–**e**) Preoperative radiographs, CT, and MRI scans showed T8–T10 vertebral bone destruction, intervertebral space narrowing and paravertebral abscess formation. (**f**,**g**) Postoperative radiographs demonstrated that internal fixation was achieved in a good position. (**h**) CT showed that solid bone fusion was obtained at 12 months after surgery. (**i**,**j**) Radiographs displayed good internal fixation position and solid bone fusion, with no loss of correction after 66 months follow-up.

The average preoperative thoracic kyphosis angle was 34.4° ± 4.5°, which decreased to 22.0° ± 2.6° postoperatively. At the final follow-up, the mean deformity angle was 23.7° ± 2.1°, with only a 1.7° ± 1.1° correction loss (p < 0.05). Single-level debridement and interbody fusion were performed in 28 patients, and two-level procedures were completed in 14 patients. Spontaneous intervertebral bone fusion was achieved 10.6 ± 2.1 months after surgery (Table 2). No nonunion, pseudoarthrosis, loosening, or breakage were observed at the final follow-up (Figs. 3 and 4).

There were no wound-related complications, except for one case of delayed incisional healing that was successfully managed with conservative treatment. Postoperative intercostal neuralgia responded to nonsteroidal anti-inflammatory drugs in five patients.

## Discussion

The global prevalence of spinal TB, the most common presentation of skeletal TB, continues to increase^15–17^. Multi-segment thoracic spinal TB is a relatively rare but very serious disease. Although medical therapy with anti-TB drugs is the mainstay of the management of tuberculous spondylitis, early surgical intervention and stabilization might be indicated to limit the risks of neurologic deficit and/or progressive instability and deformity^18,19^. The reduction of bacterial load may afford both a better response to medical therapy and the avoidance of prolonged immobilization during medical therapy alone, and may thus favor early surgical intervention^20^.

The primary aim of surgery would be simultaneous spinal stabilization and cord decompression. An anterior surgical approach has been popular due to the predominantly anterior vertebral body involvement by TB. Consequently, the anterior approach allows debridement through direct access to the infected focus^19,21,22^. However, for multi-segment thoracic spinal TB, the anterior approach used alone limits both exposure and the ability to offer stabilization across multiple diseased segments. Instability is associated with graft failure and implant breakage that predispose to cord injury. Due to this limitation, a combined anterior and posterior approach that allows stabilization and posterior instrumentation has been adopted^23,24^. However, a staged procedure requires the patient to undergo surgery twice, thus adding stress during an already nutritionally and metabolically challenged state in which TB thrives^25,26^.

With these considerations, the single-stage posterior-only approach has gained increasing acceptance for the treatment of multilevel thoracic spinal TB^27–29^. The posterior approach avoids disruption of thoracic viscera, thereby reducing surgical trauma. Furthermore, the three-column fixation of pedicle screws may provide robust biological fixation; therefore, the spine may be reconstructed immediately, and a favorable kyphosis correction may be obtained. Finally, the intervertebral defect is adequately implanted with a size-matched bone block to reconstruct the anterior column after debridement. Long-term spinal stability could follow bone graft fusion. In addition, favorable outcomes after posterior debridement and internal fixation in elderly patients with multi-segment tuberculous spondylitis have been reported^7,30^. The comparative assessments in this study supported this finding based on a quantitative analysis of the correction of kyphotic deformities, and an over 80% decrease of mean VAS scores reflecting pain intensity over the midterm post-operative follow-up interval. Significantly increased SF-36 scores suggest favorable overall health status during follow-up. These assessments demonstrated improved quality of life.

Data from this series and experience with this posterior-only approach suggest that posterior instrumentation improves biomechanical support due to its three-column fixation. This type of fixation can correct kyphosis, reduce the angle loss of deformity correction, and relieve pain due to spinal instability. Although *M. tuberculosis* does not adhere to internal fixation devices, other bacteria can adhere and form biofilms. Spinal TB was difficult to differentiate from other bacterial infections during preoperative evaluations, and was often diagnosed postoperatively. Because TB commonly involves the anterior column, a posterior approach allows internal fixation distant from the focus of infection. In addition, because TB is a chronic disease, many patients with spinal involvement are malnourished and metabolically compromised. The posterior approach reduces blood loss and anesthesia time, thereby reducing the risk of intraoperative and postoperative complications. Finally, if the mobilization of complex anatomic structures and major vessels were to be indicated through the anterior approach, the posterior approach would allow direct access and thereby minimize vascular and visceral complications.

This surgical treatment of multi-segment thoracic spinal TB can achieve posterior decompression, unilateral anterior decompression (total 270°–360° decompression), and reconstruction of anterior load support by interbody fusion that is also achieved by the thoracic reconstruction technique^31^. A thorough removal of TB lesions is the key to surgical treatment of spinal tuberculosis. Because surgery cannot achieve sterility, effective anti-TB drug treatment and improvement of the patient's general condition are important aspects of the therapy of spinal TB. Debridement may promote resolution and healing, mitigate the microenvironment that favors the survival of *M. tuberculosis*, and enhance anti-TB drug penetration into the infected focus. The removal of pus, caseous necrotic tissue, dead bone, granulation tissue, and necrotic intervertebral discs is more important than wide excision^32^. The curettage of TB lesion walls that have not sclerosed should be performed cautiously due to perilesional osteoporosis, and imaging data should be accessed intraoperatively to prevent the loss of healthy bone. Complete removal of the central lesion was also feasible for spinal canal decompression, fixation and bone grafting to confer stability. Of particular note is that bone grafting is not mandatory, as healing of diseased bone occurs with chemotherapy^33^. Satellite lesions, paravertebral pus, caseous necrotic tissue and tuberculous granulation tissue were removed and the lesions were debrided to normal bone surfaces by curettage. Paravertebral abscesses can be removed by catheter lavage, negative pressure suction and postoperative postural drainage. For patients with skip lesions, surgery is performed on segments with either severe vertebral collapse, nerve compression, or spinal instability. Lesions with slight osteolysis can be cured by conservative treatment.

We agree that the posterior-only approach might be radical and requires careful patient selection. We advocate the following considerations when managing multilevel thoracic spinal TB: (1) whenever possible and in the presence of adequate bone stock, the affected vertebrae should be incorporated into the instrumentation system; (2) enough graft should be impacted into the defect, and the intervertebral graft should be fixed to promote fusion; (3) temporary rod stabilization of the spinal cord should be provided during transforaminal thoracic debridement; (4) careful patient selection is required for this method, which is used primarily in spinal TB with limited osteolysis of the middle column; (5) careful monitoring to prevent intraoperative spinal cord injury (stretching or distraction); intraoperative spinal monitoring could be added for safety. During the entire follow-up process, all patients achieved bone fusion, and no bone nonunion or recurrence caused by insufficient debridement was observed.

This study is limited by its small size and its retrospective study design. In addition, 18 patients were lost to follow-up. However, multilevel spinal TB remains rare, and therefore, these numbers might not be disregarded. Despite the favorable outcomes of this study, the authors would recommend a larger multicenter study to compare results between the various surgical approaches before advocating a single-stage posterior approach for all patients.

## Conclusion

This midterm retrospective study of the management of multilevel thoracic spinal TB in adults showed the utility of a single-stage procedure combining posterior debridement, interbody fusion, and instrumentation; and demonstrated promising results.

## Data Availability

The datasets and materials generated or analyzed during the current study are available from the corresponding author on reasonable request.

## Abbreviations

ASIA: American Spinal Injury Association
CRP: C-reactive protein
CT: Computed tomography
ESR: Erythrocyte sedimentation rate
MRI: Magnetic resonance imaging
SF-36: 36-Item short-form
TB: Tuberculosis
VAS: Visual analog scale
